# Chemically Generated Liquid Sulfur Droplets at Room and Subzero Temperatures

**DOI:** 10.1021/acsnano.5c09160

**Published:** 2025-08-07

**Authors:** Pragadeesh Subramaniam-Venkatesh, Zhi Gao, Hongchang Hao, Xinran Xie, Tameem Karrar, Zikai Xia, Eleanor Spielman-Sun, Xia Wang, Xueli Zheng, Ankun Yang

**Affiliations:** † Department of Mechanical Engineering, 6918 Oakland University, Rochester, Michigan 48309, United States; ‡ Applied Energy Division, 497525 SLAC National Accelerator Laboratory, Menlo Park, California 94025, United States; § Stanford Synchrotron Radiation Lightsource, 497525 SLAC National Accelerator Laboratory, Menlo Park, California 94025, United States

**Keywords:** liquid sulfur, lithium iodide, lithium−sulfur, phase, redox mediator

## Abstract

The liquid phase of sulfur has been observed at room temperature, resulting from the electrochemical oxidation of polysulfides, a process occurring on the electrodes and influenced by the electrode materials. However, such electrode-dependent behavior of liquid sulfur has constrained its use in battery applications, driving research for alternative processes. This paper introduces an approach to generating liquid sulfur at both room and subzero temperatures through chemical reactions independent of the substrate material. We demonstrate that using a redox mediator, polysulfides can be chemically oxidized into liquid sulfur droplets in the electrolyte close to but away from the electrode. This pathway can generate liquid sulfur at room and subzero temperatures of −15 °C, 130 °C below sulfur’s melting temperature (115 °C). The chemically generated liquid sulfur further enriches the lithium–sulfur-electrolyte material systems, potentially creating opportunities for high-energy lithium–sulfur and other metal–sulfur batteries.

## Graphical abstract

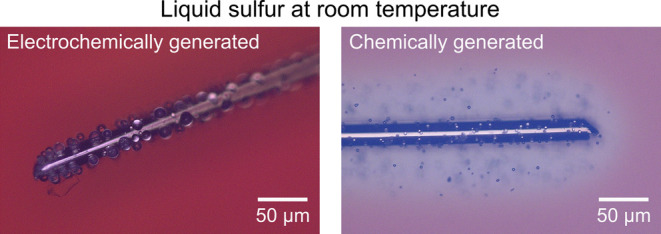

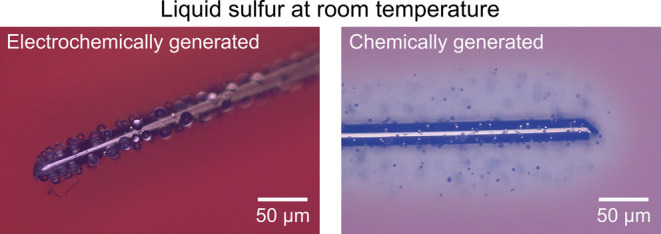

## Introduction

Sulfur is the fifth most abundant element on earth by mass and has 80 million tons annual production.^1^ Besides its notable applications in biochemical functioning, vulcanization, sulfuric acid, and fertilizer,^2–5^ sulfur is best known for its use in metal–sulfur batteries, including lithium–sulfur (Li–S),^6–10^ sodium–sulfur (Na–S),^11–13^ magnesium–sulfur (Mg–S),^14–16^ because sulfur has a specific capacity seven times higher than conventional lithium-transition metal oxide cathodes.^17^ However, the understanding of the sulfur phase and evolution under electrochemical conditions is incomplete due to the challenges in characterizing sulfur: sulfur easily sublimates under a high vacuum and gets damaged by electron beam irradiation. Using in situ optical microscopy, we recently discovered that sulfur could precipitate as a liquid phase on metallic and semiconducting substrates at and below room temperature, far below sulfur’s melting temperature of 115 °C,^18,19^ which contrasts with the traditional wisdom that sulfur remains solid during battery operations.^20^ This liquid phase of sulfur may introduce a new paradigm for energy storage applications because it has fast kinetics compared to solid sulfur; solid sulfur-based electrochemical cells generally have sluggish kinetics.^21,22^ According to our previous research and that of others,^19,23–25^ the liquid phase of sulfur, although metastable, can serve as an intermediate phase to enable fast charging of Li–S batteries. However, past research (including ours)^18,19,26^ also shows that sulfur is more likely to deposit as solid crystals instead of liquid droplets on carbon, commonly used cathode materials in metal–sulfur batteries. This significantly limits the practical impact of the liquid sulfur concept.

This paper reports the discovery of chemically generated liquid sulfur droplets at room and subzero temperatures (−15 °C) independent of substrate materials. We utilized a redox mediator, lithium iodide (LiI), to generate liquid sulfur droplets close to but away from the electrodes through two-step reactions. First, LiI is electrochemically oxidized to I_2_ on the electrodes. Second, I_2_ molecules diffuse into the electrolyte and chemically oxidize polysulfides (S_*x*_^2–^, *x* = 3–8) into liquid sulfur. This approach results in liquid sulfur droplets dispersed in the electrolyte instead of adhering to the electrodes, thus having minimal influence from the electrode materials, including widely used carbon-based electrodes. Our preliminary battery tests using carbon papers show that chemically generated liquid sulfur can significantly boost the cell capacities. The chemically generated liquid sulfur enriches the lithium–sulfur-electrolyte material systems and potentially creates opportunities for high-energy lithium–sulfur and other metal–sulfur batteries.

## Results and Discussion

Planar cells with platinum (Pt) wires as the cathode, lithium (Li) as the anode, and polysulfides in combination with various salts as the electrolytes were constructed and used for in situ electrochemical testing and simultaneous optical observations (Figure 1). With lithium bis­(trifluoromethanesulfonyl)­imide (LiTFSI) as salt in the polysulfide (S_*x*_^2–^, *x* = 3–8), liquid sulfur microsized droplets were observed over the surface of the Pt electrode at 3.5 V of constant voltage charge, showing point-contact connectivity with the wire (Figure 1a and Video S1). The generation of liquid sulfur droplets results from the electrochemical reaction: S_*x*_^2–^–2e^–^ → *x*/8 S_8_, *x* = 3–8. With continued charging, more droplets started appearing on the electrode surface. They began to fuse, forming bigger droplets. Importantly, these droplet formations were only confined to the electrode surface, which is consistent with our previous reports.^18,19,27^ Liquid sulfur droplets were consistently observed on the electrodes as the oxidation of polysulfides to sulfur required the transfer of electrons to the electrodes. Similar phenomena were observed with other salts, including lithium trifluoromethanesulfonate (LiTf) and lithium bromide (LiBr), under an applied voltage of 3.5 V (Figure S1).

**Figure 1:**
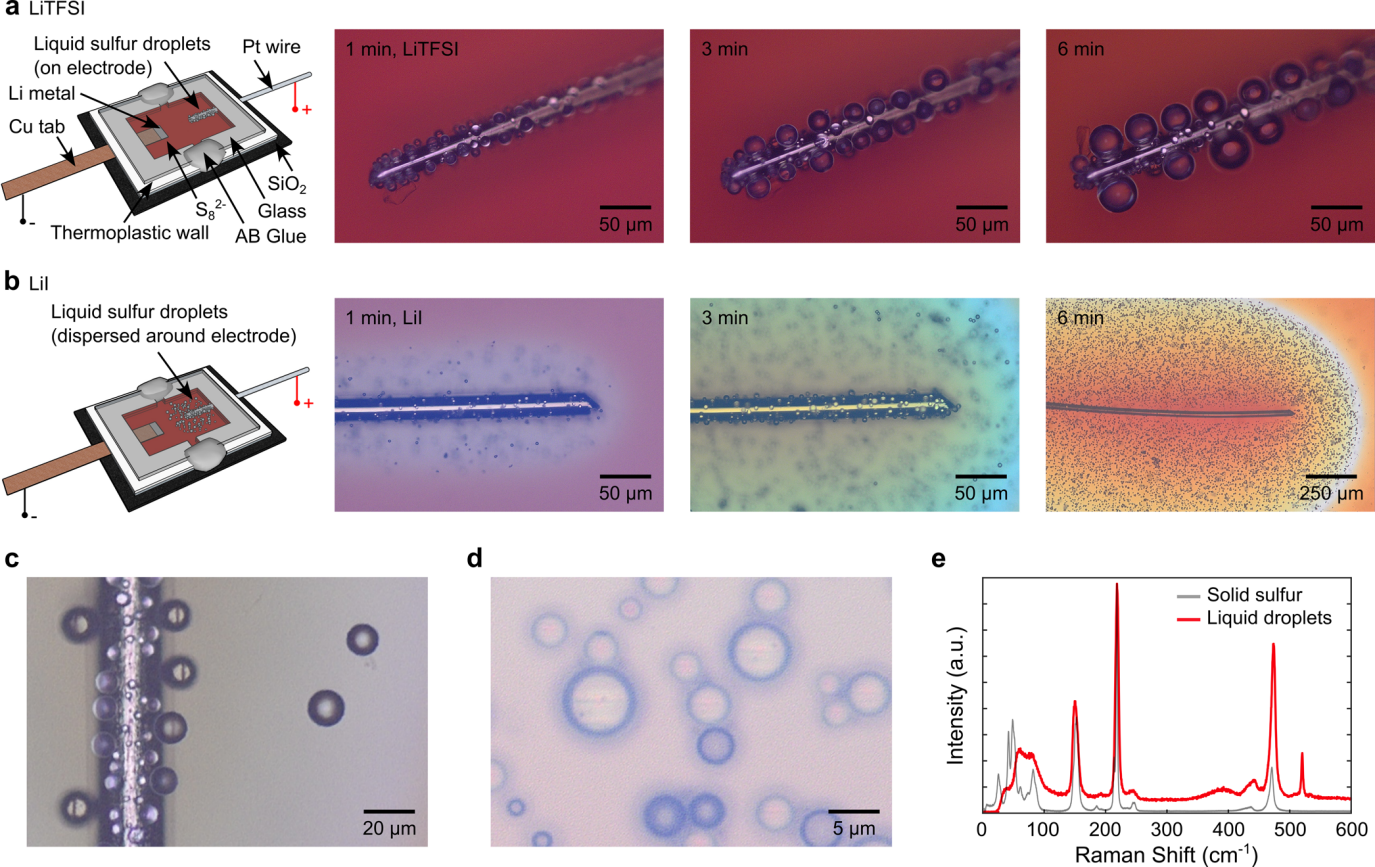
Schematic illustration and in situ optical images of liquid sulfur generation on the Pt electrode in 0.5 M polysulfide electrolyte at 3.5 V with (a) 1 M LiTFSI salt and (b) 1 M LiI salt. (c, d) A close look at the liquid sulfur droplets generated on the electrode and in the electrolyte with 1 M LiI salt. (e) The Raman spectrum of the liquid sulfur droplets in the electrolyte (red), in comparison to commercial sulfur powder (gray). We applied 3.5 V due to the higher overpotential in optical cells where the electrodes are apart from each other.

When lithium iodide (LiI) was used as the salt, instantaneous generation of liquid droplets on the electrode surface was also observed at 3.5 V, with some droplets being pushed into the electrolyte area (Figure 1b and Video S2). More interestingly, many droplets also appeared directly inside the electrolyte area away from the Pt electrode (Figure 1c). The number and density of droplets in the electrolyte area increased when the charging continued. A clear forefront boundary was observed between the droplets and the rest of the electrolyte (Figure 1b). During discharge, the droplets on the electrode surface started to dissolve first, followed by the droplets near the electrode, and then gradually by the droplets away from the electrode. There was a gradual change in the electrolyte color during the charge and discharge, and the electrolyte returned to its original color after full discharge. Similar tests on LiI were also performed at an applied constant voltage of 3.2 V (Figure S2a), where there was a noticeable drop in the droplet generation rate and quantity. At 3.0 V constant voltage, there were almost no droplets in the electrolyte area even after 5 min of charge time (Figure S2b). When focused on the droplets settled in the electrolyte area, most droplets appeared very close to each other, forming a cluster of droplets but without readily fusing to form bigger droplets (Figure 1d). This is distinctively different from the droplet fusion behavior observed with LiTFSI as salt in the polysulfides, possibly because the cluster of droplets is distributed in a 3D space. The Raman spectrum of these loose droplets exhibits typical Raman peaks for elemental S_8_ at 150, 220, and 470 cm^–1^ (Figure 1e), corresponding to molecular vibrational modes of asymmetrical S–S bending, symmetrical S–S bending, and the S–S stretching, respectively.^19,28^ This Raman spectrum resembles that of commercial sulfur powder, indicating that these droplets are sulfur in composition. The only difference in the Raman profile is noted in the low-frequency range between 0 and 100 cm^–1^, where the solid sulfur exhibits multiple phonon modes, whereas the liquid sulfur does not.^28^

Based on our in situ optical observations, we believe we have discovered chemically generated liquid sulfur at room temperature. We hypothesize that the oxidizing chemical species (I_2_, from electrochemical oxidation of the redox mediator LiI on the electrode) leads to dispersed liquid sulfur droplets in the electrolyte via the chemical oxidation of polysulfides. More specifically, there are two-step reactions. First, the LiI molecules are electrochemically oxidized to elemental iodine I_2_ (>∼3.0 V) on the electrode.^29^ Second, elemental I_2_ oxidizes polysulfides (e.g., Li_2_S_8_) to elemental S_8_ in the electrolyte. Since electrochemically generated I_2_ is free to diffuse into the electrolyte, the chemical generation of liquid sulfur droplets is not limited to the electrode surface but depends on the diffusion of I_2_.

To validate our hypothesis, we have designed a customized optical cell on a Si/Si_3_N_4_ substrate with a Si_3_N_4_ window for in situ synchrotron X-ray absorption spectroscopy (XAS) measurements (schematics: Figure 2a, photos: Figure 2b). A zoomed-in view shows that the Pt electrode was hanging on top of the Si_3_N_4_ window, which could be accessed directly from the bottom of the cell (schematic: Figure 2c, photo: Figure 2d). First, to further confirm the composition of the liquid droplets, we performed in situ sulfur K-edge X-ray absorption near-edge structure (XANES) measurements (Figure 2e). At OCV ∼2.4 V, the XANES spectrum of lithium polysulfides (Li_2_S_*x*_) exhibits two main absorption features, at 2471 eV due to the negatively charged terminal sulfur atoms and 2473 eV due to the (*x* – 2) internal atoms in the polysulfide chain.^30^ When charged to 3.5 V, the absorption feature at 2471 eV disappears, while the absorption feature at 2473 eV remains, indicating the complete conversion of S_*x*_^2–^ into elemental sulfur. The in situ sulfur K-edge XANES measurements corroborate our Raman spectroscopy results (Figure 1e), confirming that the liquid droplets in the electrolytes are composed of sulfur. Second, to confirm the existence of elemental iodine that participated in the chemical oxidation of polysulfides to sulfur, we performed in situ iodine L_3_-edge XANES measurements. At OCV ∼2.4 V, the iodine L_3_-edge XANES spectrum of lithium iodide (LiI) exhibits an absorption feature at around 4576 eV. When charged to 3.5 V, the absorption feature shifted toward high energy around 4578 eV. As the oxidation state increases, the X-ray absorption shifts to higher energy, suggesting the generation and existence of I_2_ in the electrolyte during charge.^31,32^

**Figure 2:**
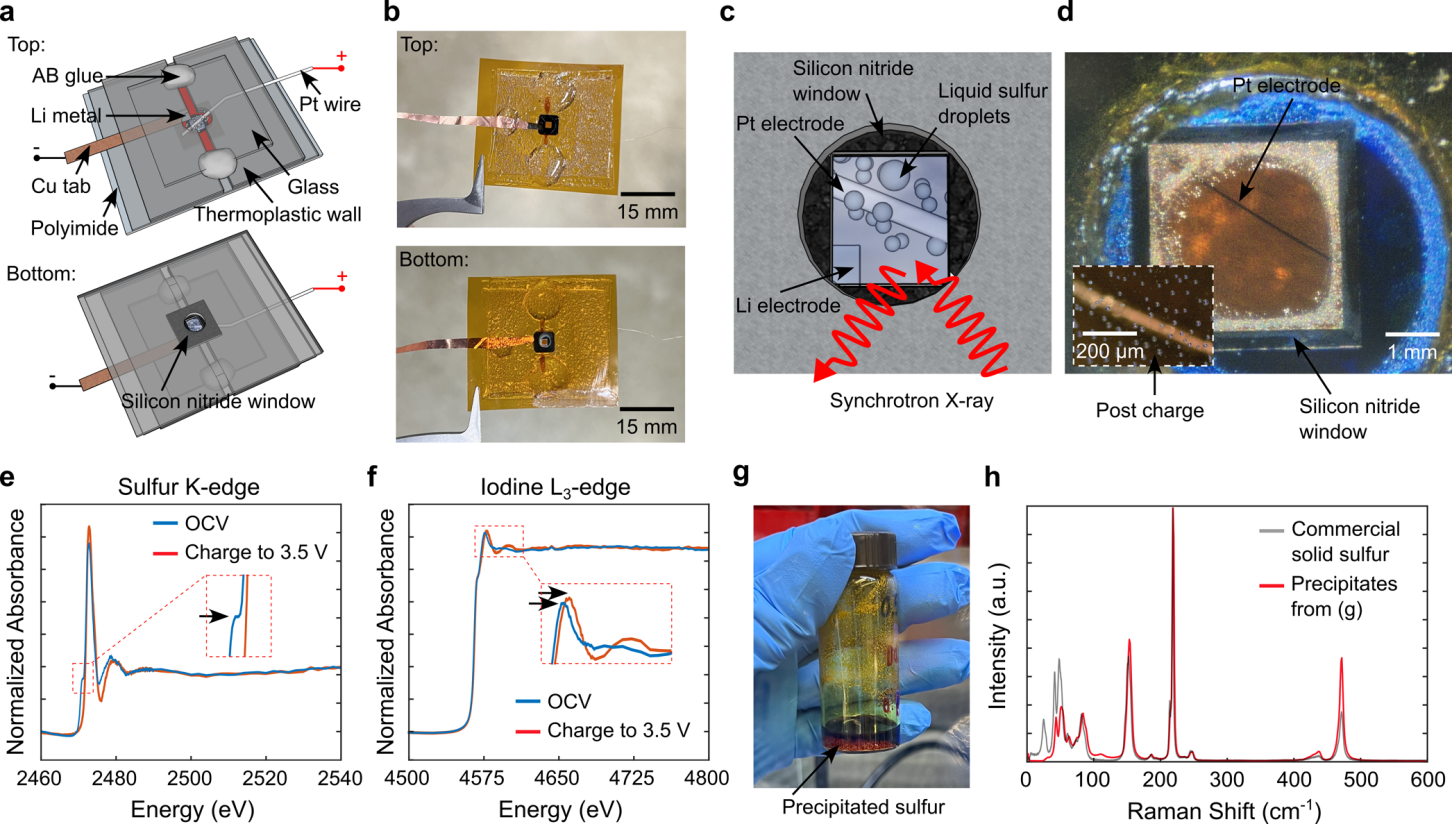
(a) Schematic and (b) photos showing the in situ XAS cell. (c) Zoomed-in schematic and (d) photo of the in situ XAS cell showing the Si_3_N_4_ window and the Pt electrode. The inset of (d) shows the chemically generated liquid sulfur droplets in the electrolyte after being charged to 3.5 V. (e) In situ sulfur K-edge XANES spectra. When charged to 3.5 V, the absorption peak at 2471 eV from negatively charged terminal sulfur atoms disappears, indicating the conversion of S_*x*_^2–^ into elemental sulfur. (f) In situ iodine L_3_-edge XANES spectra. When charged to 3.5 V, the absorption peak at 4576 eV shifts to 4578 eV, indicating the generation of elemental iodine. (g) Sulfur precipitation when a polysulfide solution was added to I_2_ in 1,3-dioxolane (DOL)/dimethyl ether (DME). (h) The Raman spectrum of the precipitates resembles that of commercial solid sulfur powder, indicating that the reaction of polysulfides and I_2_ forms sulfur.

To close the missing link, in a separate test, molecular iodine (I_2_) was dissolved in a mixture of DOL/DME to form a 1 M solution, and 1 M polysulfide was added. Instant crystallization of yellowish solids was observed (Figure 2g). These solid crystals were identified as sulfur in composition using Raman spectroscopy, consistent with that of commercially available solid sulfur (Figure 2h). Result from this test, together with those sulfur K-edge and iodine L_3_-edge XANES measurements, confirms that we have chemically generated liquid sulfur droplets at room temperature. Still, the two reaction products differ in material phase, with one being liquid sulfur droplets and the other being solid sulfur crystals. The resulting different phases might originate from the two distinct approaches to mixing the reactants. In the reaction in the glass vial (Figure 2g), I_2_ was mixed with the polysulfide solution without any fine control, and sulfur generation was violent, leading to crystallization immediately. In contrast, in the electrochemical cell, I_2_ was generated controllably and gradually before it diffused into the polysulfide solution to oxidize polysulfides into liquid sulfur droplets. This understanding suggests that the two-step reactions (electrochemical I_2_ generation + chemical S_8_ generation) have subtle control over the time and location at which the liquid sulfur droplets are generated. There have been reports of using LiI as a redox mediator in Li–S batteries, where the electrochemical oxidation of redox mediators chemically oxidizes the active material. This additional charge transfer route beyond the localized electrode interface enables homogeneous and complete oxidation of the electrode materials with a reduced overpotential.^33,34^

A piece of further evidence comes from a careful look at the LiBr salt, which has a chemical structure very similar to that of LiI. Although LiBr salt does not generate liquid sulfur droplets in the electrolyte at 3.5 V (Figure S1b), the phenomenon was noticeable at 4.5 V (Figure S3). This is because LiBr has a ∼0.5 V higher redox potential than LiI,^35^ and it requires a high voltage to be oxidized to Br, which then diffuses into the electrolyte to oxidize polysulfides to liquid sulfur droplets. Therefore, the chemical generation of liquid sulfur droplets using LiBr has a fundamental mechanism similar to that of LiI.

Given sulfur’s low electrical conductivity (5 × 10^–14^ S/m), the sulfur coating on the electrode could block electron transfer at the electrode–electrolyte interface. In contrast, if liquid sulfur droplets spread into the electrolyte, more nucleation sites are available, enlarging the effective electrode surfaces (Figure S4a,b). Consequently, more sulfur will be generated, leading to a larger capacity and faster kinetics. Therefore, we hypothesize that the salt LiI will lead to a higher capacity since I_2_ will diffuse into the electrolyte and chemically oxidize polysulfides into sulfur. To test our hypothesis, chronoamperometry has been performed on microelectrodes (size: 1–100 μm, Figure 3a) to compare the areal capacity achieved with different salts (LiTFSI vs LiI). Microelectrodes have been used in this study for the following advantages. (1) Microelectrodes with a definite surface area (e.g., an electrode with only a 25-μm disk exposed) allow us to perform a quantitative electrochemical study; (2) microelectrodes in which only the disk electrode is exposed and the remainder encapsulated in a glass capillary, minimize the likelihood of side reactions; and (3) microelectrodes allow steady-state current flow and small capacitive current, revealing true electrochemical performances. We have redesigned the preparation process for the microelectrodes based on previous reports on nanoelectrodes.^36,37^ Briefly, Ni wires (or Pt wires) attached to a tungsten (W) rod are sealed in glass capillaries using an H_2_/air flame (Figure 3b). The capillaries are then polished using silicon carbide polishing papers until a Ni disk is exposed (Figure 3c). The radius can be directly observed under an optical microscope or determined from steady-state voltammetry.^36^

**Figure 3:**
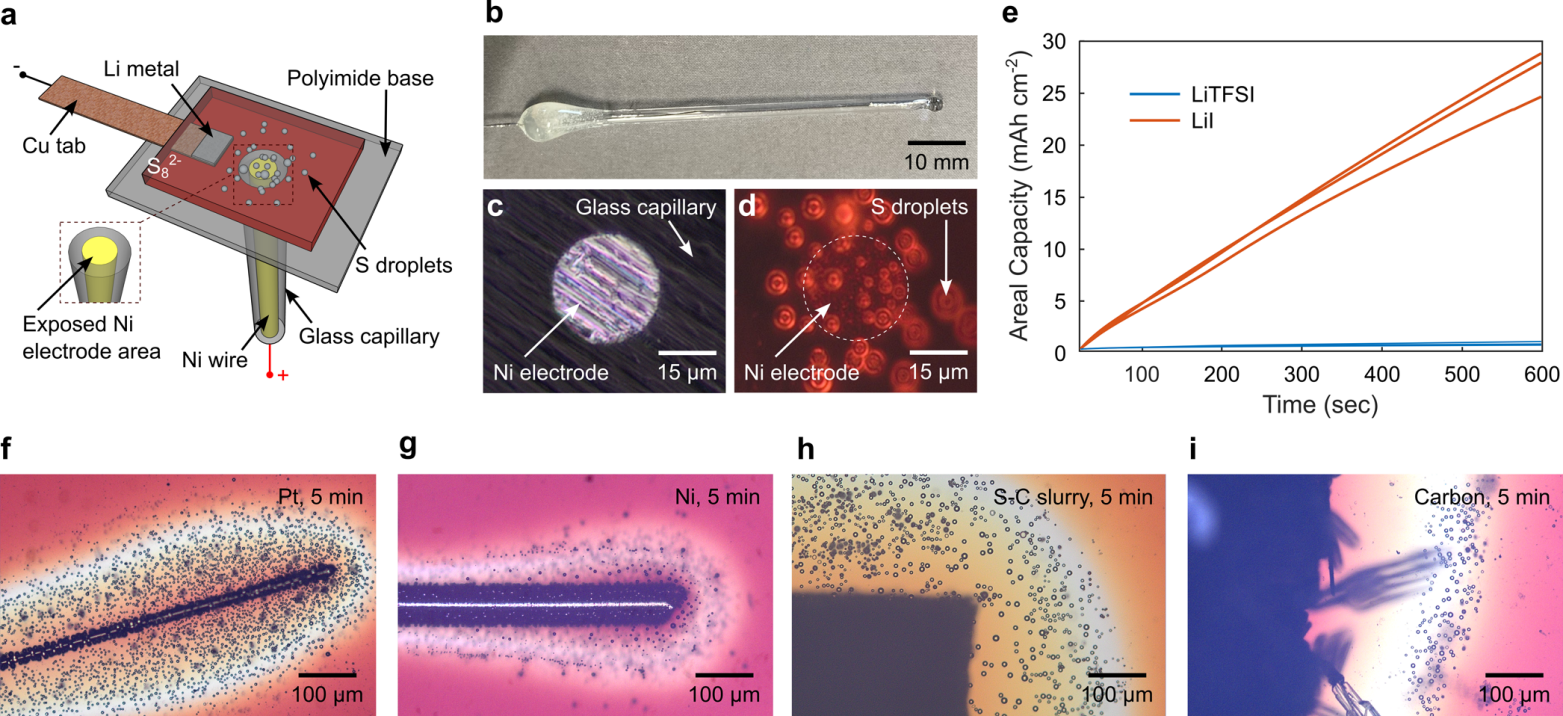
(a) Schematic of the microelectrode setup. (b) Photo of a representative microelectrode in a glass capillary. (c) A 25-μm microelectrode under the microscope. (d) Chemically generated liquid sulfur on the microelectrode. (e) Areal capacity comparison of the generated liquid sulfur with LiTFSI and LiI salts. Chemically generated liquid sulfur is independent of electrode materials: (f) platinum, (g) nickel, (h) sulfur/carbon slurry electrode, and (i) carbon paper. Note that in (e), part of the capacity came from the LiI/I_2_ redox reaction; however, the generated I_2_ contributed to the final sulfur capacity.

With LiTFSI, the areal capacity had an average value of ∼0.6 mAh/cm^2^ in 10 min at 3.5 V. The trend was comparable in the electrodes tested with LiTFSI and almost overlapped in the plot (Figure 3e). In contrast, with LiI as salt, a significant increase in areal capacity to ∼26 mAh/cm^2^ was observed, and the trend was consistent across all electrodes measured. The quantification was consistent with our visual observations of the generated dispersed liquid sulfur droplets with LiI salt, which were not limited to the microelectrode area (Figure 3d). The observation of many more liquid sulfur droplets explains the high charge capacity. The oxidation of LiI to I_2_ can also contribute to the capacity, and a portion of I_2_ was likely diffusing out of the electrode and contributing toward the chemical oxidation of polysulfides in the electrolyte. This additional charge transfer route allows the generation of more sulfur droplets, not limited to the electrode interfaces. We also tested the rates of liquid sulfur generation as a function of salts directly using Pt wires, and the results showed a pattern similar to the areal capacity from LiI, which was much higher than that from LiTFSI (Figure S4c).

Certain substrate materials, including carbon electrodes, can promote heterogeneous nucleation with a lower energy barrier, leading to sulfur crystallization.^18^ When sulfur molecules are formed in the electrolyte away from the carbon electrodes, we hypothesize that the substrate material’s role in controlling the sulfur phase will be reduced, and the observed liquid phase of sulfur will essentially be independent of the substrate material. To test this hypothesis, we screened different electrode materials for chemically generating liquid sulfur, including Pt, Ni, sulfur/carbon slurry electrodes, and carbon paper. We observed a consistent generation of liquid sulfur droplets in the electrolyte close to but away from the electrodes (Figure 3f–i). The similar spread of liquid droplets in the electrolyte region suggests that chemically generated liquid sulfur is not dependent on the type of electrode material. Even though sulfur crystallization was observed over the carbon electrode (Figure 3i), liquid sulfur droplet generation was observed in the electrolyte area. This phenomenon suggests that a rational design of carbon electrodes and the electrolyte (e.g., using appropriate redox mediators) can (1) generate liquid sulfur droplets in the electrolyte for fast charging and high capacity (which also facilitates recovering sulfur from migrating polysulfides) and (2) collect liquid sulfur through solid crystal crystallization for the discharge. The conversion of metastable liquid sulfur into stable solid crystals was observed in our previous study,^18^ and was confirmed with carbon fiber as a cathode material in our studies (Figure S5). This opens up exciting opportunities for utilizing this phenomenon in carbon-based electrodes commonly used in metal–sulfur batteries.

The electrochemistry was tested in coin cells with dried carbon paper as the cathode, lithium metal as the anode, and polysulfides as the catholyte. Cyclic voltammetry (CV) was performed on a cell with 1 M LiI salt dissolved in 5 M polysulfide until 3.2 V, and it was observed that the LiI gets oxidized to I_2_ at about 2.95 V during the charge process (Figure 4a). This is the potential at which I_2_ is generated, which triggers the chemical generation of liquid sulfur. During discharge, I_2_ is reduced to LiI at about 2.85 V. Figure 4b shows a comparison of performance when these cells were charged to 2.8 and 3.2 V. Charging to 3.2 V enables the oxidation of LiI to I_2_, which then chemically oxidize polysulfides to sulfur leading to the gain of additional charge capacity as shown in the orange plot (Figure 4b), reaching ∼810 mAh/g overall. During discharge, we have observed an offset plateau (γ) in the plot before the regular discharge plateaus for the Li–S systems, which was due to the reduction of remaining I_2_ back to LiI. This offset amounts to ∼70 mAh/g, and the overall discharge capacity reaches ∼760 mAh/g. Using the regular charge/discharge curves (charge to 2.8 V, discharge to 1.8 V, blue plot) as the reference (discharge capacity ∼ 640 mAh/g), the overall gain in discharge capacity after the offset due to I_2_ reduction is about 50 mAh/g (δ).

**Figure 4:**
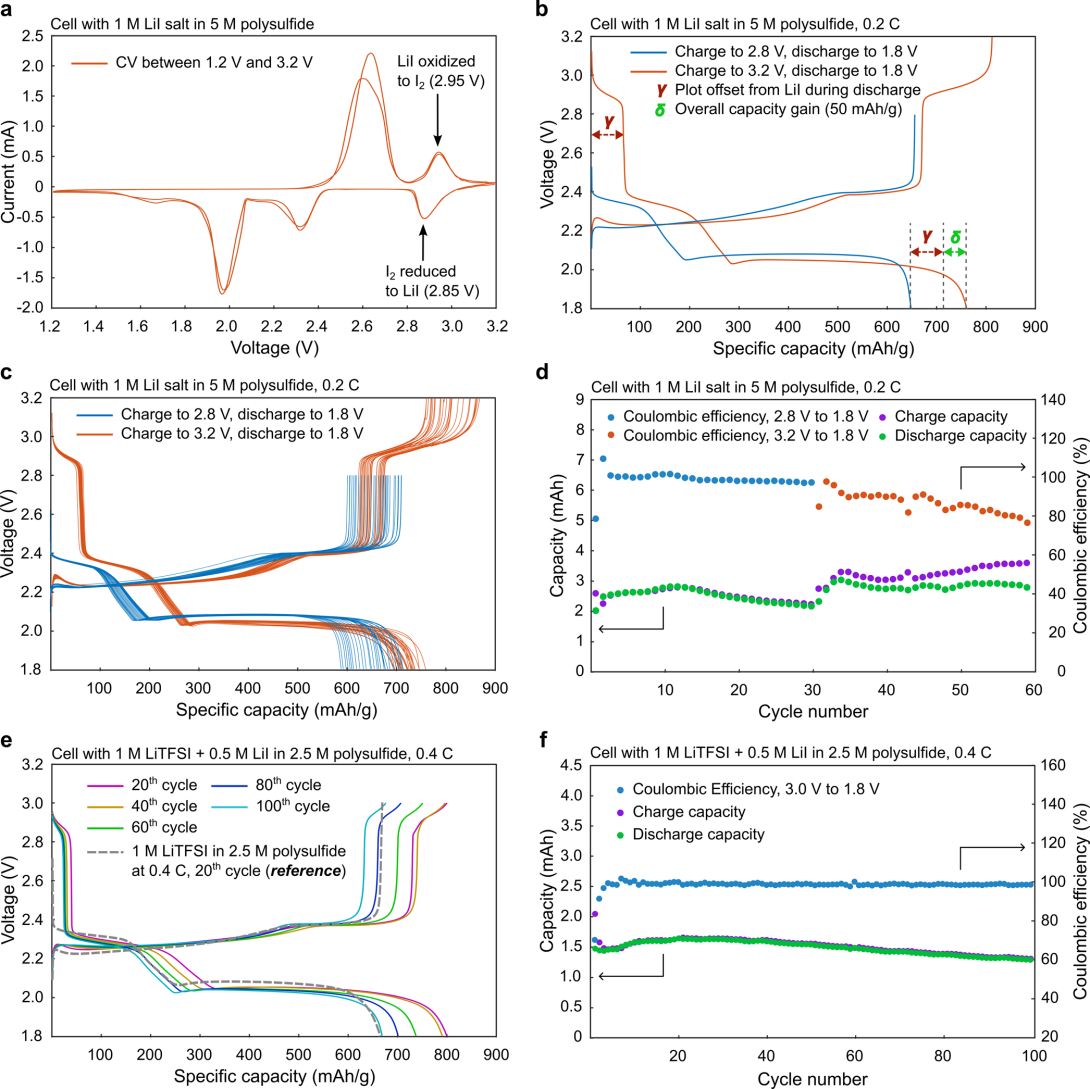
(a) CV of a coin cell with 1 M LiI in 5 M polysulfide as a catholyte showing the oxidation of LiI to I_2_ at 2.95 V. (b) Comparison of coin cell charge–discharge cycles to an upper limit of 2.8 vs 3.2 V and the associated discharge capacity gain after subtracting the plot offset due to I_2_ reduction. (c) 30 consecutive charge–discharge cycles to an upper limit of 2.8 and 3.2 V. (d) Capacity and Coulombic efficiency for 30 cycles at 2.8 V max and 30 cycles at 3.2 V max. (e) Cells with 1 M LiTFSI and 0.5 M LiI in 2.5 M polysulfide cycled at 0.4 C to 3.0 V compared with 1 M LiTFSI in 2.5 M polysulfide at 0.4 C as a reference in dashed line. (f) Capacity and Coulombic efficiency for the cell shown in panel (e) for 100 consecutive cycles.

We performed 30 consecutive charge–discharge cycles between 1.8 and 2.8 V, followed by 30 cycles between 1.8 and 3.2 V at 0.2 C (Figure 4c,d). The plots show that both the charge and discharge capacity increase when the cells were charged to 3.2 V. As seen in Figure 4d, the Coulombic efficiency is stable for the first 30 cycles when the cycling was limited between 1.8 and 2.8 V; however, the Coulombic efficiency was relatively low for the 3.2 V charge. This may be attributed to the fact that some liquid sulfur droplets remain in the electrolyte area after the end of the discharge process (at 1.8 V), bringing the overall discharge capacity down. Although we have demonstrated the conversion of liquid sulfur into a solid and its reconnection to the electrode (Figure S5), this process depends on the precise engineering of the carbon electrode structure. Rationally designed carbon electrodes with optimized porosity and pore size may address this issue, which is our ongoing work. To improve the cycle life, an electrolyte with a combination of salts (1 M LiTFSI + 0.5 M LiI) in 2.5 M polysulfide was studied, as LiTFSI has proven to provide stable cycling and excellent capacity retention, in combination with LiI for capacity boost. This time, the upper limit of the charge cycle was brought down to 3.0 V to be more amenable to practical applications, and the charge/discharge C-rate was increased to 0.4 C. The resulting specific capacity drop after 100 cycles with a combination of LiTFSI and LiI salts (0.4 C, 3.0 V upper limit) was equivalent to the drop recorded after 30 cycles with LiI (0.2 C, 3.2 V upper limit). The dashed line in Figure 4e was from a reference cell with only 1 M LiTFSI in 2.5 M polysulfide at 0.4 C at the 20th cycle. It is evident from this comparison that the cell with a combination of salts has a significantly higher capacity (800 mAh/g; ∼750 mAh/g after deducting the offset due to I_2_ reduction) compared to the cell with only LiTFSI salt (680 mAh/g). Importantly, charging to only 3.0 V would still generate liquid sulfur, resulting in a corresponding capacity gain. The Coulombic efficiency has also been improved significantly with the combination of salts and remained at ∼100% throughout the 100 cycles (Figure 4f).

Sulfur-based electrochemistry is well-known to work at low temperatures.^10^ We have previously shown that liquid sulfur can be electrochemically generated at subzero temperatures as low as −28 °C.^18^ Here, we attempted to probe whether liquid sulfur can be chemically generated at subzero temperatures. Since the chemical generation of liquid sulfur droplets in electrolytes involves the diffusion of I_2_, which is related to the temperature via the diffusion coefficient:

$$D=D_{0}e^{\left(-Q_{d}/\mathrm{RT}\right)}$$

where *D*_0_ is pre-exponential, *Q*_d_ is the activation energy, *R* is the gas constant, and *T* is the temperature. The diffusion coefficient *D* decreases with decreasing temperature, which indicates that the diffusion of I_2_ will be limited at low temperatures. Experiments at subzero temperatures were conducted by placing the optical battery cells with LiI salt on a thermal stage that is capable of controlling temperature between −196 °C (liquid nitrogen) and 600 °C (Figure 5a). After 5 min of idle soak at −15 °C to reach thermal equilibrium, a constant voltage of 3.5 V was applied, and liquid sulfur droplets were found to appear on and in the close vicinity of the electrode (Figure 5b). With time, the density of the chemically generated liquid sulfur droplets in the electrolyte grew, but at a relatively slower rate than those generated at room temperature (Figure 1b).

**Figure 5:**
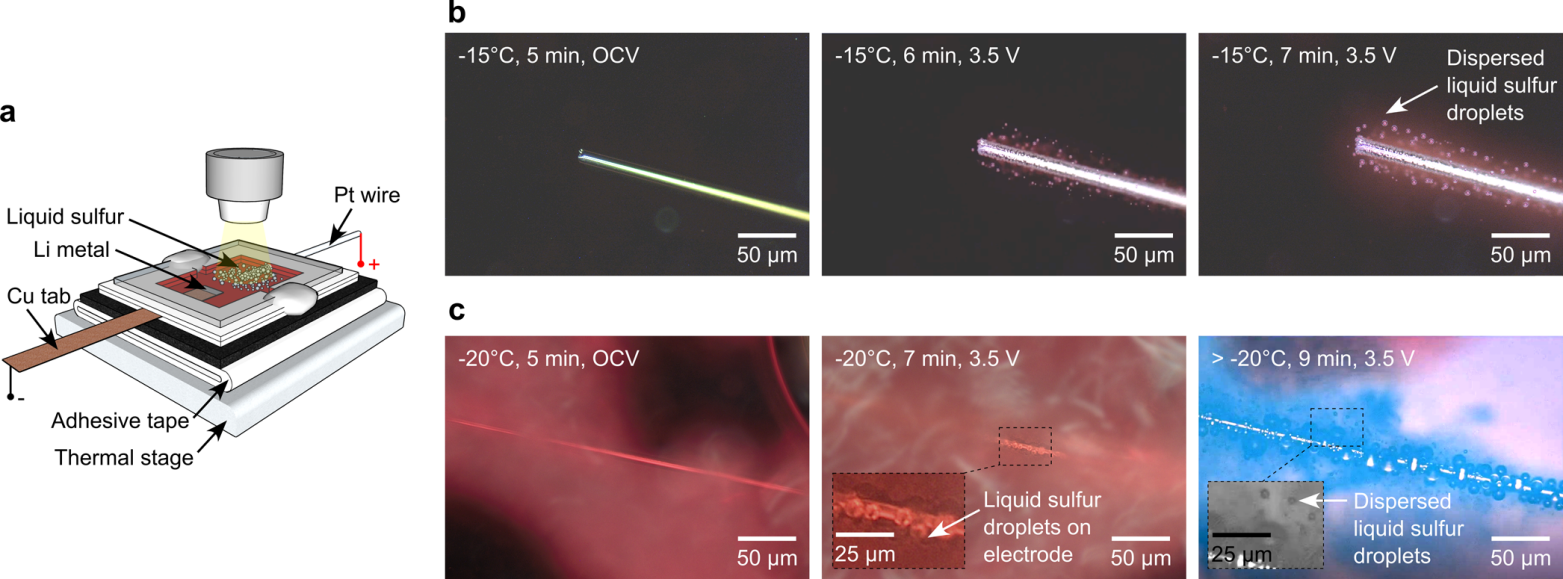
(a) Schematic illustration of the subzero temperature test setup with the optical cell positioned on a thermal stage. Microscopic images of liquid sulfur generation and progression on a Pt electrode immersed in polysulfide with LiI salt when the cell temperature is (b) −15 °C and (c) −20 °C (the cloudy species on the glass surface in panel (c) was from water condensation at low temperatures).

When the experiment was performed at −20 °C, the liquid sulfur droplets started to appear only on the surface of the Pt electrode after applying 3.5 V (Figure 5c). No droplets were observed in the electrolyte. We then slowly ramped up the cell temperature and observed the initiation of the liquid sulfur droplets in the electrolyte area (Figure 5c, inset). This indicates that I_2_ has slower diffusion kinetics at subzero temperatures, limiting the generation of liquid sulfur droplets in the electrolyte. The lowest temperature at which we could observe chemically generated liquid sulfur droplets in this study was −15 °C.

## Conclusions

In this study, we report chemically generated liquid sulfur droplets at both room and subzero temperatures for the first time. We demonstrate that by utilizing a redox mediator (e.g., LiI), we can implement a two-step reaction process where the redox mediator is first electrochemically oxidized, followed by the chemical oxidation of polysulfides into the liquid phase of sulfur. Importantly, the chemical generation of liquid sulfur is independent of substrate materials, which creates many opportunities, including the application of liquid sulfur in commonly used carbon-based electrodes. We have performed preliminary tests implementing the idea of chemically generated liquid sulfur to boost the cell capacity. Additionally, we show that liquid sulfur droplets can be chemically generated at subzero temperatures as low as −15 °C. These findings enrich the lithium–sulfur-electrolyte material systems and may facilitate the rational design and broader utilization of liquid sulfur in practical lithium–sulfur and other metal–sulfur batteries.

## Materials and Methods

### Preparation of the Electrolytes

Polysulfide (Li_2_S_8_) was prepared by chemically reacting lithium sulfide (Li_2_S) with sulfur in a 1:1 volume mixture of dimethyl ether (DME) and 1,3-dioxolane (DOL). This mixture was then stirred overnight at around 50 °C in an argon-filled glovebox (H_2_O, O_2_ <0.5 ppm) until all of the solids were completely dissolved to synthesize a reddish-brown polysulfide electrolyte solution. For optical cell tests, 1 M LiTFSI, LiI, LiTf, or LiBr was dissolved in the 0.5 M polysulfide to form the final electrolytes. For coin cell tests, 5 or 2.5 M polysulfides with LiTFSI and/or LiI were used, along with 2 wt % of lithium nitrate (LiNO_3_). For the mixed salt configuration, 1 M LiTFSI and 0.5 M LiI salts, along with 2 wt % of LiNO_3_, were added to a mixture of 1:1 DOL/DME until the salts fully dissolved. An equivalent quantity of 5 M polysulfide was added to this mixture to bring the overall polysulfide concentration to 2.5 M. This order was followed to ensure complete dissolution of salts.

### Preparation of the Optical Cell

Platinum (Pt) wire with a diameter of 25 μm was used as the cathode. Lithium (Li) metal laminated on a copper current collector tab was used as the anode. The optical cell was assembled with a silicon dioxide wafer as a base, the Li-coated copper tab on one side, and the Pt electrode on the other side, with a cover glass on top for in situ observations. A thermoplastic film was used to seal the optical cell, leaving two small openings for filling the electrolyte. The polysulfide electrolyte with different salts was injected into the optical cell to wet and connect both electrodes. After electrolyte filling, epoxy was used to seal the two openings. This completed optical cell was then transferred out of the glovebox for further testing. For the XAS optical cell, a silicon wafer with a silicon nitride window was used as the base. A piece of polyimide film was used to extend the base area for easy assembly.

### In Situ Testing of the Optical Cell at Room Temperature

At room temperature, the optical cell was positioned under the microscope (Olympus, 20 or 50×), and the Pt wire was focused. The anode and cathode tabs of the cell were connected to a BioLogic SP-150e potentiostat to follow a charge–discharge sequence. A 3.5 V constant voltage was applied across the cell to charge for 10 min while the cathode surface area and the surrounding electrolyte area were continuously monitored under the microscope. The cell was then galvanostatically discharged at 0.02 mA. Planar battery cells with side-by-side arrangement of the cathode and anode generally have a higher internal impedance, so relatively higher voltage (e.g., 3.5 V) is applied compared to other forms of battery cells, such as coin cells (e.g., 2.8 V).

### In Situ Testing of the Optical Cell at Subzero Temperature

A Linkam thermal stage (−196 to 600 °C) was used to control the temperature of the optical cell with a thermally conductive adhesive tape on the stage surface to hold the optical cell. In this configuration, we performed control experiments for the temperature sensed on a piece of silicon dioxide wafer. Based on the control experiments, all of the temperatures reported were after correction. Nitrogen fumes were continuously applied on top of the optical cell glass slide to reduce moisture condensation at subzero temperatures, thereby enabling clear viewing through the microscope. Once the thermal stage reached subzero temperatures, the optical cell was allowed to settle at that temperature for 5 min. An infrared thermometer was used to measure and monitor the surface temperature of the optical cell. A 3.5 V constant voltage was applied via BioLogic SP-150e potentiostat across the cell to charge for 10 min when the Linkam stage temperature was held constant. The cathode surface and surrounding electrolyte areas were monitored under the microscope.

### In Situ Raman Spectroscopy

In situ Raman spectroscopy was performed using a Horiba LabRAM Raman spectrometer with 532 nm laser excitation, 600 grooves/mm grating, and a 50× long working distance objective.

### X-ray Absorption Near-Edge Structure (XANES) Spectroscopy

Sulfur K-edge and iodine L_3_-edge XANES measurements were conducted at beamline 14-3 of the Stanford Synchrotron Radiation Lightsource (SSRL) at the SLAC National Accelerator Laboratory. All data were collected in fluorescence mode using a 7-element vortex detector using an air-exclusion chamber purged with He gas located in the sample-to-detector path. The monochromator was calibrated by setting the maximum energy of the pre-edge feature of sodium thiosulfate to 2472.02 eV. Measurements were made using a Si(111) double-crystal monochromator. Spectra were recorded using a beam spot size of 1 μm in diameter. XANES spectra were normalized, and the background was subtracted using Demeter Athena software.

### Coin Cell Assembly and Testing

Carbon paper (Fuel Cell Store) was preheated in a lab oven and then transferred into a glovebox and heated again on a hot plate to remove any moisture residues. Carbon paper was cut and used as the cathode current collector. 25 μL of catholyte (polysulfides with salt) was applied using a pipet on the carbon cathode. The Celgard 2400 separator was then placed on the catholyte-soaked electrode. Then, 25 μL of anolyte (salt + 2 wt % LiNO_3_ + DOL/DME) was added on the separator. The molar concentration of the salts differs and was noted in the main text. Finally, a precut Li disc was placed on the separator after removing the oxidation layer. The coin cell was crimped to complete the assembly. These coin cells are moved to a LAND battery cycler to apply a charge–discharge program at a C-rate of choice to assess the performance. Cyclic voltammetry (CV) measurements were performed using a CHI potentiostat between 1.2 and 3.2 V with a scan rate of 0.1 mV/s.

## Supplementary Material







## Data Availability

The data generated in the study are available from the corresponding author upon reasonable request.

## Notes

In situ optical observations of liquid sulfur generation with different salts, including LiTf, LiBr, and LiI, at different voltages; areal capacity comparison of the generated liquid sulfur with LiTFSI and LiI salts; and optical observation of the phase transition of chemically generated liquid sulfur into solid (PDF)
Liquid sulfur droplet generation with 1 M LiTFSI at 3.5 V (Video S1) (MP4)
Liquid sulfur droplet generation with 1 M LiI at 3.5 V (Video S2) (MP4)

## References

[1] U.S. Geological Survey Mineral Commodity Summaries . Sulfur, 2022. https://pubs.usgs.gov/periodicals/mcs2022/mcs2022-sulfur.pdf.

[2] Beinert H. (2000). A tribute
to sulfur. Eur. J. Biochem..

[3] Krejsa M. R., Koenig J. L. (1993). A Review of Sulfur
Crosslinking Fundamentals for Accelerated
and Unaccelerated Vulcanization. Rubber Chem.
Technol..

[4] King, M. ; Moats, M. ; Davenport, W. G. Sulfuric Acid Manufacture: Analysis, Control and Optimization. Elsevier, 2nd ed.; 2013.

[5] Boswell C. C., Friesen D. K. (1993). Elemental sulfur fertilizers and their use on crops
and pastures. Fert. Res..

[6] Bruce P. G., Freunberger S. A., Hardwick L. J., Tarascon J.-M. (2012). Li–O2 And
Li–S Batteries with High Energy Storage. Nat. Mater..

[7] Ji X., Lee K. T., Nazar L. F. (2009). A highly
ordered nanostructured carbon–sulphur
cathode for lithium–sulphur batteries. Nat. Mater..

[8] Yang Y., Zheng G., Cui Y. (2013). A Membrane-Free
Lithium/Polysulfide
Semi-Liquid Battery for Large-Scale Energy Storage. Energy Environ. Sci..

[9] Jin Y., Zhou G., Shi F., Zhuo D., Zhao J., Liu K., Liu Y., Zu C., Chen W., Zhang R., Huang X., Cui Y. (2017). Reactivation of dead sulfide species
in lithium polysulfide flow battery for grid scale energy storage. Nat. Commun..

[10] Xu S., Zhang L., Zhang H., Wei M., Guo X., Zhang S. (2020). A high-energy, low-temperature lithium-sulfur
flow battery enabled
by an amphiphilic-functionalized suspension catholyte. Mater. Today Energy.

[11] Yang F., Mousavie S. M. A., Oh T. K., Yang T., Lu Y., Farley C., Bodnar R. J., Niu L., Qiao R., Li Z. (2018). Sodium–Sulfur Flow Battery for Low-Cost Electrical Storage. Adv. Energy Mater..

[12] Wang Y., Zhang Y., Cheng H., Ni Z., Wang Y., Xia G., Li X., Zeng X. (2021). Research Progress
toward Room Temperature
Sodium Sulfur Batteries: A Review. Molecules.

[13] Wei S., Xu S., Agrawral A., Choudhury S., Lu Y., Tu Z., Ma L., Archer L. A. (2016). A stable room-temperature sodium–sulfur battery. Nat. Commun..

[14] Kim H. S., Arthur T. S., Allred G. D., Zajicek J., Newman J. G., Rodnyansky A. E., Oliver A. G., Boggess W. C., Muldoon J. (2011). Structure
and compatibility of a magnesium electrolyte with a sulphur cathode. Nat. Commun..

[15] Gao T., Noked M., Pearse A. J., Gillette E., Fan X., Zhu Y., Luo C., Suo L., Schroeder M. A., Xu K., Lee S. B., Rubloff G. W., Wang C. (2015). Enhancing the Reversibility
of Mg/S Battery Chemistry through Li+ Mediation. J. Am. Chem. Soc..

[16] Gao T., Hou S., Wang F., Ma Z., Li X., Xu K., Wang C. (2017). Reversible S0/MgSx Redox Chemistry in a MgTFSI2/MgCl2/DME Electrolyte
for Rechargeable Mg/S Batteries. Angew. Chem.,
Int. Ed..

[17] Zhou G., Chen H., Cui Y. (2022). Formulating energy density for designing
practical lithium–sulfur batteries. Nat.
Energy.

[18] Liu N., Zhou G., Yang A., Yu X., Shi F., Sun J., Zhang J., Liu B., Wu C.-L., Tao X., Sun Y., Cui Y., Chu S. (2019). Direct Electrochemical Generation
of Supercooled Sulfur Microdroplets Well Below Their Melting Temperature. Proc. Natl. Acad. Sci. U.S.A..

[19] Yang A., Zhou G., Kong X., Vila R. A., Pei A., Wu Y., Yu X., Zheng X., Wu C.-L., Liu B., Chen H., Xu Y., Chen D., Li Y., Fakra S., Hwang H. Y., Qin J., Chu S., Cui Y. (2020). Electrochemical generation of liquid
and solid sulfur on two-dimensional
layered materials with distinct areal capacities. Nat. Nanotechnol..

[20] Dibden J. W., Smith J. W., Zhou N., Garcia-Araez N., Owen J. R. (2016). Predicting the composition and formation of solid products
in lithium–sulfur batteries by using an experimental phase
diagram. Chem. Commun..

[21] Zhang M., Chen W., Xue L., Jiao Y., Lei T., Chu J., Huang J., Gong C., Yan C., Yan Y., Hu Y., Wang X., Xiong J. (2020). Adsorption-Catalysis Design in the
Lithium-Sulfur Battery. Adv. Energy Mater..

[22] Liu F., Sun G., Wu H. B., Chen G., Xu D., Mo R., Shen L., Li X., Ma S., Tao R., Li X., Tan X., Xu B., Wang G., Dunn B. S., Sautet P., Lu Y. (2020). Dual redox mediators accelerate the
electrochemical kinetics of lithium-sulfur batteries. Nat. Commun..

[23] Zhou G., Yang A., Gao G., Yu X., Xu J., Liu C., Ye Y., Pei A., Wu Y., Peng Y., Li Y., Liang Z., Liu K., Wang L.-W., Cui Y. (2020). Supercooled
liquid sulfur maintained in three-dimensional current collector for
high-performance Li-S batteries. Sci. Adv..

[24] Shi F., Guo X., Chen C., Zhuang L., Yu J., Qi Q., Zhu Y., Xu Z.-L., Lau S. P. (2023). Unlocking Liquid
Sulfur Chemistry
for Fast-Charging Lithium–Sulfur Batteries. Nano Lett..

[25] Shi F., Chen C., Qi Q., Meng R., Zhuang L., Yang Z., Liu D., Xu Z.-L., Lau S. P. (2024). Optimal
liquid sulfur deposition dynamics for fast-charging Li-S batteries. Energy Storage Mater..

[26] Shi F., Onofrio N., Chen C., Cai S., Li Y., Zhai L., Zhuang L., Xu Z.-L., Lau S. P. (2022). Stable
Liquid-Sulfur Generation on Transition-Metal Dichalcogenides toward
Low-Temperature Lithium–Sulfur Batteries. ACS Nano.

[27] Zhou G., Yang A., Wang Y., Gao G., Pei A., Yu X., Zhu Y., Zong L., Liu B., Xu J., Liu N., Zhang J., Li Y., Wang L.-W., Hwang H. Y., Brongersma M. L., Chu S., Cui Y. (2020). Electrotunable liquid
sulfur microdroplets. Nat. Commun..

[28] Nims C., Cron B., Wetherington M., Macalady J., Cosmidis J. (2019). Low Frequency
Raman Spectroscopy for Micron-Scale and in Vivo Characterization of
Elemental Sulfur in Microbial Samples. Sci.
Rep..

[29] Wu F., Lee J. T., Nitta N., Kim H., Borodin O., Yushin G. (2015). Lithium Iodide as a Promising Electrolyte
Additive
for Lithium–Sulfur Batteries: Mechanisms of Performance Enhancement. Adv. Mater..

[30] Pascal T. A., Wujcik K. H., Velasco-Velez J., Wu C., Teran A. A., Kapilashrami M., Cabana J., Guo J., Salmeron M., Balsara N., Prendergast D. (2014). X-ray Absorption Spectra of Dissolved
Polysulfides in Lithium–Sulfur Batteries from First-Principles. J. Phys. Chem. Lett..

[31] Zhou J., Chandrappa M. L. H., Tan S., Wang S., Wu C., Nguyen H., Wang C., Liu H., Yu S., Miller Q. R. S., Hyun G., Holoubek J., Hong J., Xiao Y., Soulen C., Fan Z., Fullerton E. E., Brooks C. J., Wang C., Clément R. J., Yao Y., Hu E., Ong S. P., Liu P. (2024). Healable and conductive
sulfur iodide for solid-state Li–S batteries. Nature.

[32] Zhang S., Zhao F., Su H., Zhong Y., Liang J., Chen J., Zheng M. L., Liu J., Chang L., Fu J., Alahakoon S. H., Hu Y., Liu Y., Huang Y., Tu J., Sham T., Sun X. (2024). Cubic Iodide LixYI3+x Superionic
Conductors through Defect Manipulation for All-Solid-State Li Batteries. Angew. Chem., Int. Ed..

[33] Gao X., Zheng X., Tsao Y., Zhang P., Xiao X., Ye Y., Li J., Yang Y., Xu R., Bao Z., Cui Y. (2021). All-Solid-State
Lithium–Sulfur Batteries Enhanced by Redox
Mediators. J. Am. Chem. Soc..

[34] Tsao Y., Lee M., Miller E. C., Gao G., Park J., Chen S., Katsumata T., Tran H., Wang L.-W., Toney M. F., Cui Y., Bao Z. (2019). Designing a Quinone-Based Redox Mediator to Facilitate
Li2S Oxidation in Li-S Batteries. Joule.

[35] Kwak W.-J., Hirshberg D., Sharon D., Afri M., Frimer A. A., Jung H.-G., Aurbach D., Sun Y.-K. (2016). Li–O 2 cells
with LiBr as an electrolyte and a redox mediator. Energy Environ. Sci..

[36] Zhang B., Galusha J., Shiozawa P. G., Wang G., Bergren A. J., Jones R. M., White R. J., Ervin E. N., Cauley C. C., White H. S. (2007). Bench-Top Method for Fabricating Glass-Sealed Nanodisk
Electrodes, Glass Nanopore Electrodes, and Glass Nanopore Membranes
of Controlled Size. Anal. Chem..

[37] Luo L., White H. S. (2013). Electrogeneration
of Single Nanobubbles at Sub-50-nm-Radius
Platinum Nanodisk Electrodes. Langmuir.

