# Supplementary material for: Chemically Generated Liquid Sulfur Droplets at Room and Subzero Temperatures
Source: ACS Nano. 2025 Aug 7;19(32):29682–90. doi: 10.1021/acsnano.5c09160 (PMC12369013; doi:10.1021/acsnano.5c09160)
Supplement: Supplementary file 1 [file nn5c09160_si_001.pdf]

Supporting Information

Chemically Generated Liquid Sulfur Droplets at Room and Subzero Temperatures

Pragadeesh Subramaniam-Venkatesh[1], Zhi Gao[1], Hongchang Hao[2], Xinran Xie[1], Tameem Karrar[1], Zikai Xia[1], Eleanor Spielman-Sun[3], Xia Wang[1], Xueli Zheng[2], and Ankun Yang[1,]*

1. Department of Mechanical Engineering, Oakland University, Rochester, MI 48309, USA

2. Applied Energy Division, SLAC National Accelerator Laboratory, Menlo Park, California 94025, USA

3. Stanford Synchrotron Radiation Lightsource, SLAC National Accelerator Laboratory, Menlo Park, California 94025, USA

***Corresponding author. Email: ankunyang@oakland.edu (A.Y.)**

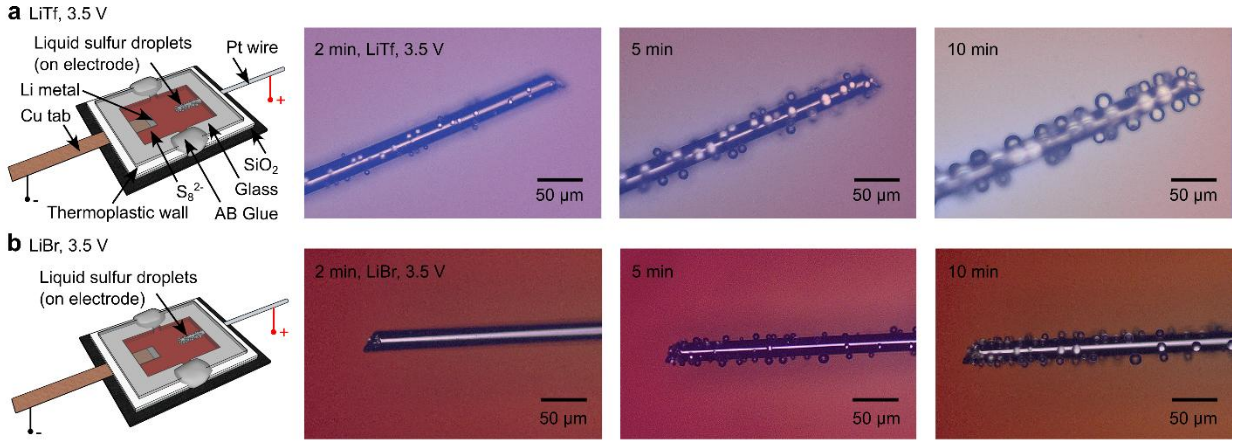

Fig. S1 Schematic illustration and microscopic images of liquid sulfur generation in (a) 1 M lithium trifluoromethanesulfonate (LiTf) salt in 0.5 M polysulfide electrolyte on Pt electrode at an applied voltage of 3.5 V, (b) 1 M lithium bromide (LiBr) salt in 0.5 M polysulfide electrolyte on Pt electrode at an applied voltage of 3.5 V.

Observations with lithium trifluoromethanesulfonate (LiTf) were similar to those from LiTFSI (Fig. 1a), with sulfur droplets appearing on the electrode surface and increasing in size as charging progressed. With lithium bromide (LiBr), droplet nucleation was comparatively slower, resulting in smaller droplet sizes by the end of the 10-minute charge cycle. No droplets were seen in the electrolyte area with either salt at 3.5 V.

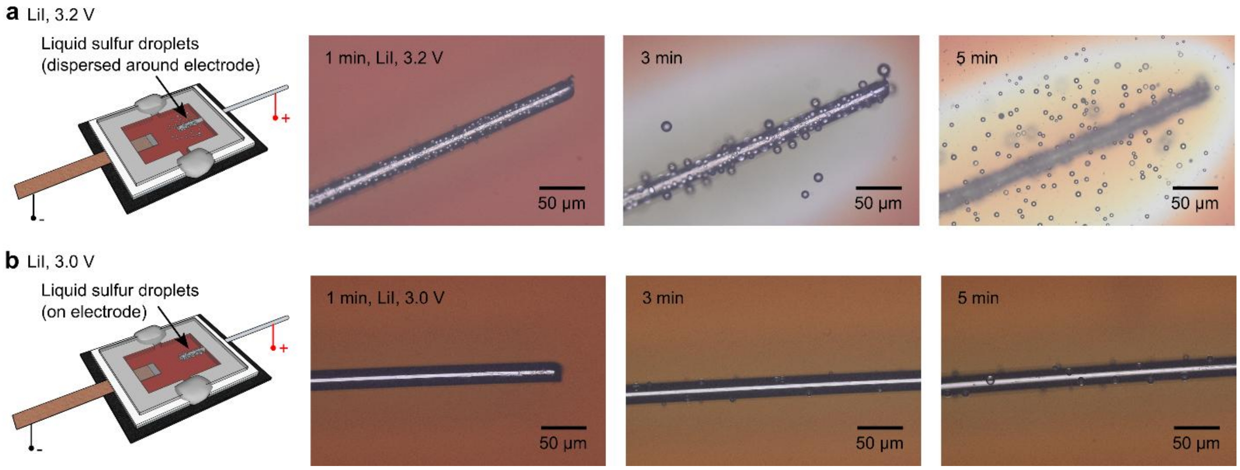

Fig. S2 Schematic illustration and microscopic images of liquid sulfur generation with 1 M LiI salt in 0.5 M polysulfide electrolyte on Pt electrode at (a) 3.2 V and (b) 3.0 V.

At an applied voltage of 3.2 V, immediate droplet generation was observed (**Fig. S2a**), with continued charging droplets also seen in the electrolyte area, and the density increased steadily. A key difference at 3.2 V compared with charging at 3.5 V (Fig. 1b) was the relatively low rate of droplet formation in the electrolyte area and lower droplet density. However, no immediate droplet generation was observed at an applied voltage of 3.0 V with 1 M LiI salt in polysulfide (**Fig. S2b**). After about 3 minutes of constant voltage charging at 3.0 V, some droplets are observed over the surface of the platinum wire. With continued charging for about 5 minutes, more droplets appeared on the electrode surface, but no droplets were in the electrolyte area.

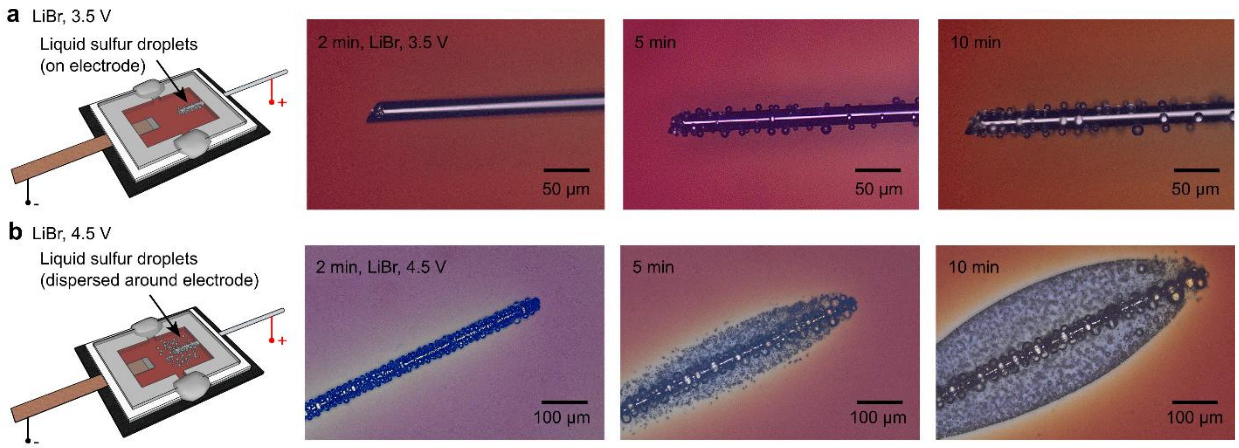

Fig. S3 Schematic illustration and microscopic images of liquid sulfur generation in 1 M LiBr salt in 0.5 M polysulfide electrolyte on Pt electrode at an applied voltage of (a) 3.5 V and (b) 4.5 V.

LiBr salt at an applied voltage of 3.5 V exhibited behavior similar to LiTFSI (Fig. 1a), with the sulfur droplets only appearing on the electrode surface even after 10 minutes of charge time (**Fig. S3a**). However, at an applied voltage of 4.5 V, we began to observe droplet formation in the electrolyte area, similar to that of LiI salts (Fig. 1b). The droplet density and area occupied in the electrolyte increased with the charge time (**Fig. S3b**). We achieved chemically generated liquid sulfur with LiBr salt at a higher voltage because LiBr has ~0.5 V higher redox potential than LiI.[1] We note that at 4.5 V, side reactions occurred in the cell, and the electrolyte began to degrade. This experiment demonstrated that we could generate liquid sulfur droplets using LiBr as a redox mediator; however, this process may not be suitable for practical applications due to its high voltage.

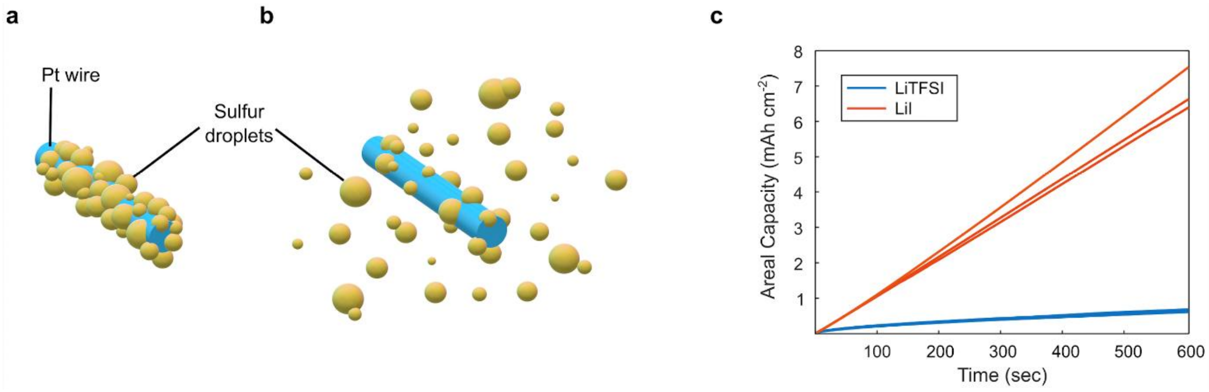

Fig. S4 Schematic showing liquid sulfur generation patterns with different salts: (a) LiTFSI, and (b) LiI. (c) Areal capacity comparison of the generated liquid sulfur with LiTFSI and LiI salts. Note that in (b), part of the capacity came from LiI/I_2 redox reaction; however, the generated I_2 contributed to the final sulfur capacity.

Three Pt wire electrodes with comparatively different exposed areas were tested with each salt type. Areal capacity was calculated by dividing the measured charge capacity by the exposed Pt electrode area in the electrolyte. With LiTFSI, the areal capacity was ~ 0.8 mAh cm^{-2} in 10 minutes at 3.5 V. The trend was comparable in all three electrodes tested with LiTFSI and almost overlapped in the plot. In contrast, with LiI as the salt, a significant increase in the areal capacity of ~ 7 mAh cm^{-2} was observed, and the trend was consistent across all three electrodes measured. It is interesting to note that the areal capacity with LiTFSI salt from the Pt wire (~ 0.8 mAh cm^{-2}) was close to that from the Ni microelectrode (~ 0.6 mAh cm^{-2}, Fig. 3e), while the areal capacity with LiI salt from the Pt wire (~ 7 mAh cm^{-2}) was much lower than that from the Ni microelectrode (~ 26 mAh cm^{-2}, Fig. 3e). This is because Pt wires were lying on top of the Si/SiO_2 wafer, limiting the diffusion of the generated I_2 into 3D space from the electrode surface. In contrast, the microelectrodes have no such limitation. Nonetheless, in both tests, the LiI salt will lead to much higher areal capacity compared to the LiTFSI salt, due to the chemically generated liquid sulfur droplets.

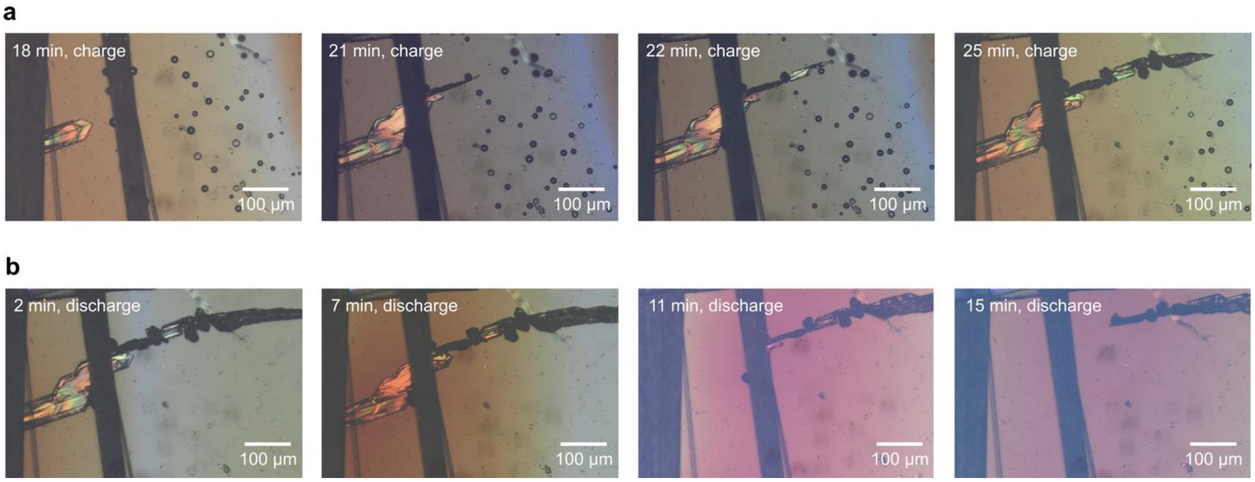

Fig. S5 (a) Microscopic images of crystal growth from the carbon fiber cathode while consuming liquid sulfur droplets generated in the electrolyte area during the charge process. (b) Microscopic images of sulfur crystal dissolution during discharge.

Optical cells with carbon fibers as the cathode were prepared and tested with 1 M LiI dissolved in 0.5 M polysulfides as the electrolyte. Chemically generated liquid sulfur droplets were observed in the electrolyte area after charging. After ~ 18 minutes, solid crystal sulfur growth was observed starting from carbon fibers and continuing to grow as charging progressed. These crystals started to convert the liquid sulfur droplets into crystals, consuming them to grow into bigger crystals. During discharge, the sulfur crystal close to the carbon fibers began dissolving first into polysulfide, as evident from the color change at approximately 7 minutes, and this pattern continued as discharge progressed.

Supplementary Videos

Supplementary Video 1: Liquid sulfur droplet generation with 1 M LiTFSI at 3.5 V. Speed x24.

Supplementary Video 2: Liquid sulfur droplet generation with 1 M LiI at 3.5 V. Speed x8.

References

1. Kwak, W.-J., Hirshberg, D., Sharon, D., Afri, M., Frimer, A. A., Jung, H.-G., Aurbach, D. & Sun, Y.-K. Li–O_2 cells with LiBr as an electrolyte and a redox mediator. *Energy Environ. Sci.* **9,** 2334–2345 (2016).
